# Medullary sponge kidney: unusual finding in kidney transplant recipient

**DOI:** 10.1186/s13089-022-00277-3

**Published:** 2022-09-29

**Authors:** M. Righini, C. Felicani, A. Lopez, E. Mazzotta, C. De Molo, E. Mancini, I. Capelli, C. Serra, G. La Manna

**Affiliations:** 1 Nephrology and Dialysis Unit, Santa Maria Delle Croci Hospital, Ravenna, Italy; 2 Nephrology, Dialysis and Renal Transplant Unit, IRCCS–Azienda Ospedaliero-Universitaria di Bologna, Alma Mater Studiorum, University of Bologna, Bologna, Italy; 3 Internal Medicine Unit, Azienda Ospedaliero Universitaria Di Modena, Modena, Italy; 4 Division of Nephrology and Dialysis, Malpighi Hospital, Bologna, Italy; 5 Internal Medicine Unit, Sant’Orsola-Malpighi Hospital, Bologna, Bologna, Italy; 6 Interventional Ultrasound Unit, Department of Organ Failure and Transplantations, Sant’Orsola-Malpighi Hospital and University of Bologna, Bologna, Italy

**Keywords:** Medullary sponge kidney, Kidney transplantation, Sonography

## Abstract

**Background:**

Medullary sponge kidney is generally considered a benign condition, gold standard for the diagnosis is urography but it has almost been replaced by UroCT that did not present the same sensibility. Although it is really rare, our sonography’s findings were consistent with medullary sponge kidney in the transplanted kidneys.

**Case presentation:**

A 45-year-old woman with a long history of double-kidney transplantation complained of frequent urinary tract infections, a history of vague loin pain and came to our attention for sonography follow-up. Her kidney function was normal, we did not find signs of infections in the transplanted kidneys and urinary findings were normal. Curiously, the transplanted kidneys came from a newborn and the patient received a double-kidney transplantation in order to guarantee a satisfactory renal function.

**Conclusions:**

Despite a long history of kidney transplantation, genetic disease should not be forgotten when symptoms and images recall to specific inherited alterations. Sonography has to be considered in diagnostic path of kidney cystic disease.

## Introduction

Medullary sponge kidney (MSK) is a kidney malformation that normally occurs in one of the numerous steps characterizing renal morphogenesis. Abnormalities in genes fundamental for renal formation lead to diminished distal nephron development, causing cyst formation, nephrocalcinosis and distal renal tubular acidosis as subsequent consequence of urine concentration defects [1]. Dilatation of the collecting ducts in the renal medulla or in renal papillae produce the spongy appearance for which the disease is named [2]. MSK is generally considered a sporadic disorder, but an apparently autosomal dominant inheritance has also been observed. The disease affects both genders, with a slight female prevalence, and is generally diagnosed in adulthood (20–30 years old). Clinical manifestations include nephrolithiasis, urinary tract infections, micro- or macro-hematuria, hypercalciuria and hypocitraturia, but it is worth saying that often it may be asymptomatic and it is diagnosed as an incidental finding during diagnostic follow-up [3]. When it becomes manifest in children, the disease is more severe and is accompanied by rickets-like symptoms. Recurrent calcium nephrolithiasis and nephrocalcinosis are the most common signs, hyperparathyroidism is frequently associated.

Although the pathogenesis of MSK is yet to be determined, the idea that it is a developmental disorder is supported by its association with different malformative conditions (Wilms tumor, Horse-shoe kidney, autosomal dominant polycystic kidney disease, other urinary tract malformations) especially with Beckwith–Wiedemann syndrome [4, 5].

The diagnosis of MSK is made through intravenous urography (gold standard), but nowadays it has almost been replaced by computed tomography urography (CTU) even though it did not present the same sensibility. Concerning ultrasound, MSK presents a peculiar tetrad: hypoechoic medullary areas, hyperechoic spots, microcystic dilatations of papillary zone and multiple calcifications (linear, small stones or calcified intracystic sediment) in each papilla that, added to laboratory data and clinical history, could be helpful to identify patients with MSK [6]. Though differential diagnosis for cystic renal disease in “young kidneys” is very challenging, Thomas et al. proposed a pragmatic reporting format for cystic renal diseases [7]. The most common differential diagnosis are with nephronophthisis (NPH) and/or medullary cystic kidney disease (ADTKD). In NPH, kidneys are small to normal in size, with increased echogenicity, reduced cortico-medullary differentiation, and renal cysts formation on the cortico-medullary border. In ADTKD, kidneys are normal to small in size, with multiple cysts at the cortico-medullary junction and sometimes in the renal medulla, but renal stones and microcalcifications are not usually detected in these cases [8].

Aside from recurrent episodes of nephrolithiasis, MSK can be considered a benign condition. Most treatment regimens are centered around prophylactic measures compared to symptomatic care and a proper therapy for stones formations [9]. Considering organ shortages, Cheungpasitporn W and colleagues aimed to assess the outcomes of living kidney donors with MSK, reporting the safety of MSK kidney donors with normal kidney function, low kidney stone risk and no significant comorbidity [10].

## Case presentation

We present a case of a 45-year-old female, who received a double-kidney transplantation 29 years before our evaluation. She came to our attention in the context of her normal transplant follow-up. Medical history reported in 1983 diagnosis of nephronophthisis, 1990 peritoneal dialysis, 1991 total parathyroidectomy for severe hyperparathyroidism, 1991 double renal transplantation, 2002 tibia’s bone dystrophy. The patient reports several urinary tract infections in the last 2 years, and several episodes of vague loin pain without a radiological or biochemical correlation. In the latest sonographies, was reported that kidneys presented millimetrical hyperechoic spots and multiple calcifications in transplanted organs.

Laboratory findings: renal function was normal (creatinine 0,91 mg/dl, BUN 32 mg/dl, eGFR 84 ml/min), calcium 9,1 mg/dl, parathormone (PTH) was undetectable, calciuria and citraturia were normal, no bacterial were founded in urine or blood.

**Fig. 1:**
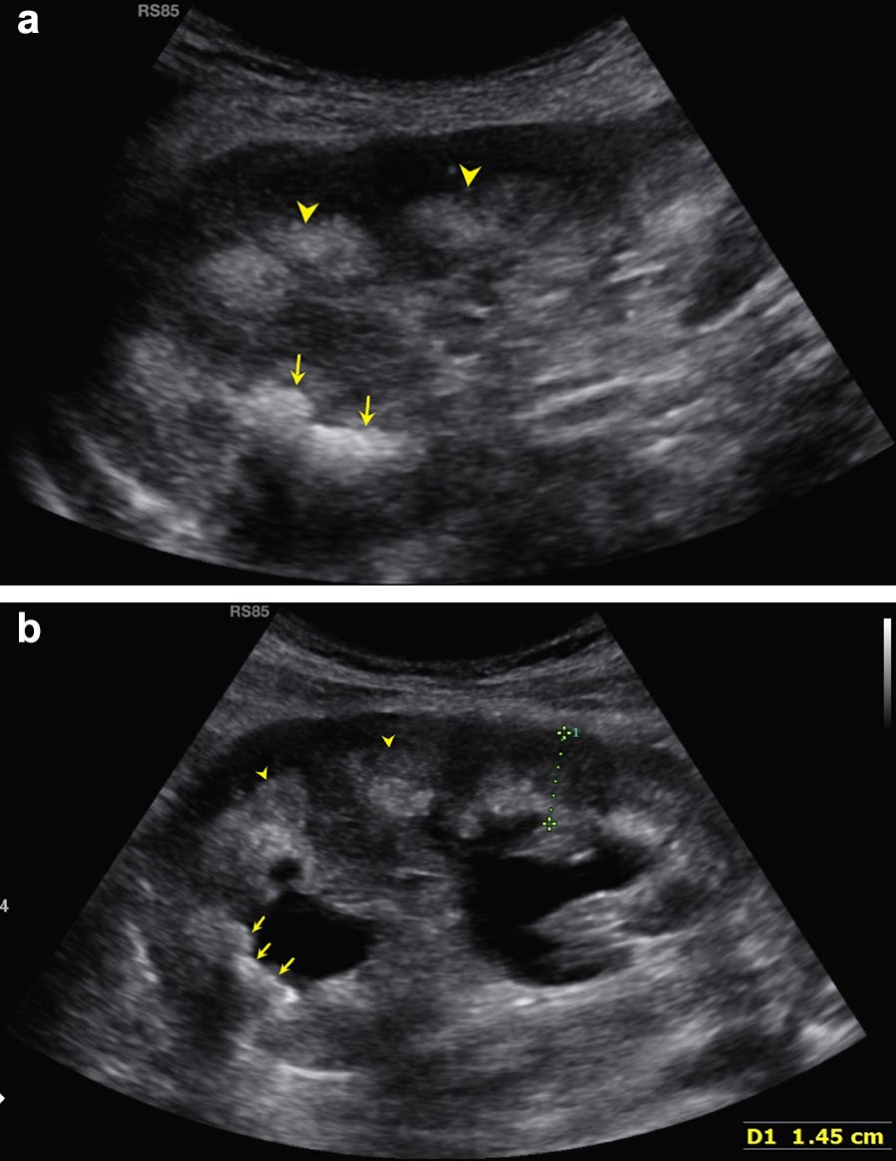
Philips EPIQ, convex transductor, B-mode, frequency 2,5–5 MHz, right lower abdomen quadrant. **a** The caudal kidney shows a hypoechoic cortical with hyperechoic medulla (arrowheads). The superior calyceal group presents even large kidney stones with the typical rear shadow cone behind (arrows). **b** The cranial kidney shows the same echographics characteristics of the caudal kidney: a hypoechoic cortical with hyperechoic medulla (arrowheads), and kidney stones with rear shadow cone (arrows). Moreover, we can appreciate the dilatation of renal pelvis probably due to bladder replenish or urethral stones. Renal parenchymal thickness (among calipers) is normal suggesting a preserved renal parenchyma. Both kidneys present the same sonography pattern, consistent with medullary sponge kidney (MSK)

**Fig. 2:**
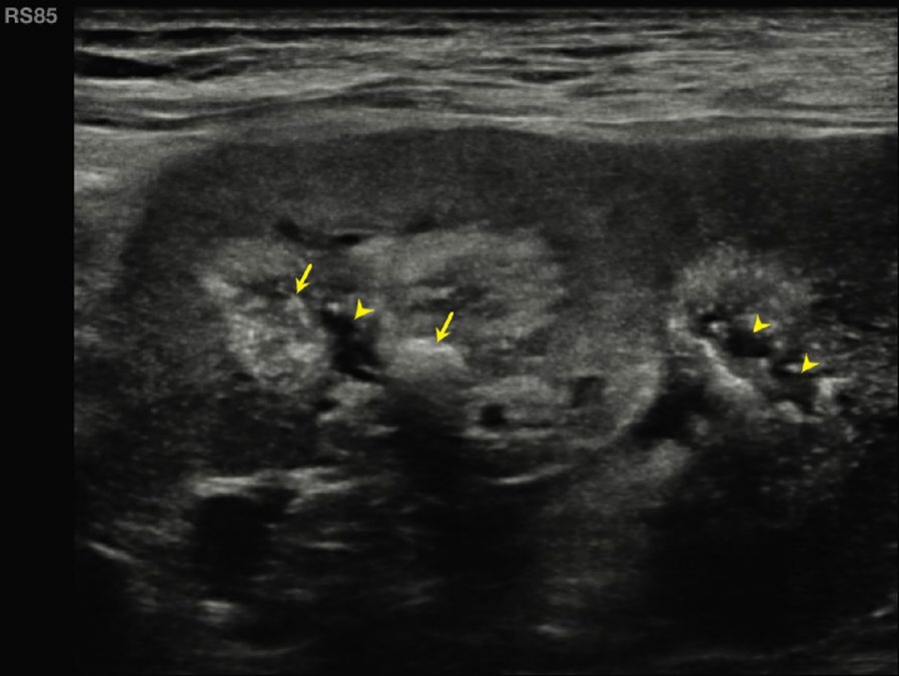
Philips EPIQ, linear transductor, B-mode, frequency 5–12 MHz, right lower abdomen quadrant, cranial kidney: with a highly detailed linear probe it is possible to appreciate in the cranial kidney, recognizable for the pelvic dilatation, the presence of microcystic anechoic dilatations in papillary zone (signed by yellow arrowheads) and multiple diffuse hyperechoic spots (calcifications, signed by yellow arrows)

**Fig. 3:**
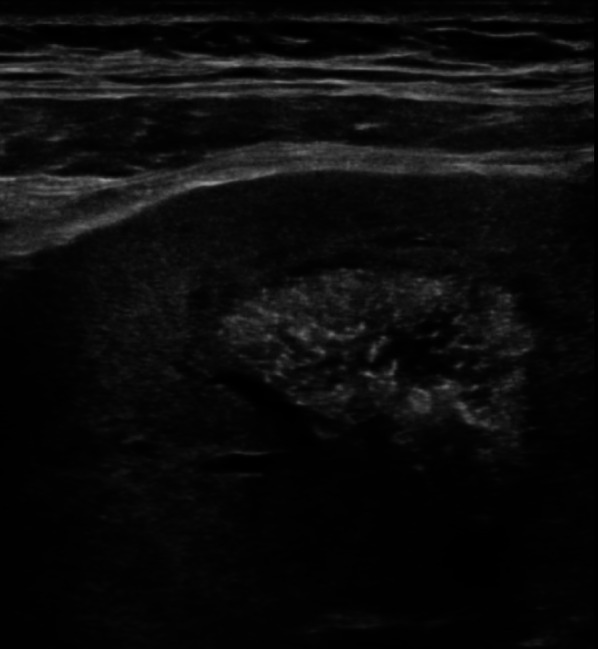
Philips EPIQ, linear transductor, B-mode, frequency 5–12 MHz, right lower abdominal quadrant, detail: another detail of the typical aspect of renal papilla. Linear high-resolution probe shows millimetric hyperechoic spots with a rear shadow cone in the papilla, highly suggestive for calcifications, a typical alteration of medullary sponge kidney

We performed abdomen ultrasound that showed wrinkled native kidneys, though transplanted kidneys were localized both in the right iliac fossa, presented normal size (10 cm and 9,5 cm), cortical diameter was normal (14 mm and 15 mm), diffuse hyperechoic medullary (Fig. 1) with some hypoechoic areas (consistent with microcystic dilatation of papillary zone), several diffuse millimetrical hyperechoic spots (from 1–2 mm to 7 mm) in each papillae, consistent with calcifications (Figs. 2 and 3). The cranial transplanted kidney shown a second degree hydronephrosis in the context of replenish bladder, without distal obstructions (Fig. 1b).

Ultrasound findings are consistent with medullary sponge kidney in the transplanted kidneys.

Curiously, the transplanted kidneys came from a newborn (< 1 year) and the size mismatch obliged the clinicians to perform a double-kidney transplantation, in order to guarantee satisfactory renal function.

## Case point

The patient complained of loin pain and often about dysuria, she presented normal renal function and no urine abnormalities. Sonography is highly suggestive of MSK, but finding this anomaly in transplanted kidneys is something really rare. Considering the long story of kidney transplant, it could not be ethically proposed to perform UroCT in a patient with normal renal function, due to the high potential risk of contrast-induced nephropathy, and according to a few studies, magnetic resonance (MR) is not sensitive enough to disclose the typical signs of MSK. Considering that the donor was a newborn, it would have been difficult to detect those kind of alterations at the moment of transplantation, therefore it is technically possible that this female inherited two kidneys with these anomalies. To our knowledge, no cases of double transplantation with MSK in a deceased donor have been previously reported. There may be several explanation: first, the low prevalence of the disease; second, the long-term presentation of the disease that normally exceeds transplant survival; third, the mostly asymptomatic course of the disease; fourth, the paucisymptomatic course of kidney stones in the transplanted kidney; last, the difficulty to detect this kind of disease.

## Conclusions

MSK is a rare genetic kidney disease that remains paucisymptomatic for a long time, though it could have been found in a kidney transplantation. It is at risk of being underdiagnosed in the near future because urography is less and less used in the diagnostic work-up, but sonography supported by clinics, history and biochemical analysis could help establish the diagnosis of MSK.

## Data Availability

The datasets used and/or analyzed during the current study are available from the corresponding author on reasonable request.

## Abbreviations

ADTKD: Autosomal dominant tubulointerstitial kidney disease
BUN: Blood urea nitrogen
CTU: Computer tomography urography
eGFR: Estimated glomerular filtration rate
MR: Magnetic resonance
MSK: Medullary sponge kidney
NPH: Nephronophthisis
PTH: Parathormone

## References

[1] Imam TH, Patail H, Patail H (2019). Medullary sponge kidney: current perspectives. Int J Nephrol Renovasc Dis.

[2] Xiang H, Han J, Ridley WE, Ridley LJ (2018). Medullary sponge kidney. J Med Imaging Radiat Oncol.

[3] Fabris A, Anglani F, Lupo A, Gambaro G (2013). Medullary sponge kidney: state of the art. Nephrol Dial Transplant.

[4] Choyke PL, Siegel MJ, Oz O (1998). Nonmalignant renal disease in pediatric patients with Beckwith-Wiedemann syndrome. Am J Roentgen.

[5] Gambaro G, Feltrin GP, Lupo A, Bonfante L, D’Angelo A, Antonello A (2006). Medullary sponge kidney (Lenarduzzi-Cacchi-Ricci disease): a Padua Medical School discovery in the 1930s. Kidney Int.

[6] Pisani I, Giacosa R, Giuliotti S, Moretto D, Regolisti G, Cantarelli C, Vaglio A, Fiaccadori E, Manenti L (2020). Ultrasound to address medullary sponge kidney: a retrospective study. BMC Nephrol.

[7] Thomas CC, Jana M, Sinha A, Bagga A, Ramachandran A, Sudhakaran D (2021). Ultrasound imaging of renal cysts in children. J Ultrasound Med.

[8] Katabathina VS, Kota G, Dasyam AK, Shanbhogue AK, Prasad SR (2010). Adult renal cystic disease: a genetic, biological, and developmental primer. Radiographics.

[9] Gambaro G, Danza FM, Fabris A (2013). Medullary sponge kidney. Curr Opin Nephrol Hypertens.

[10] Cheungpasitporn W, Thongprayoon C, Brabec BA, Kittanamongkolchai W, Erickson SB (2016). Outcomes of living donors with medullary sponge kidney. Clin Kidney J.

